# PHI-base – the multi-species pathogen–host interaction database in 2025

**DOI:** 10.1093/nar/gkae1084

**Published:** 2024-11-26

**Authors:** Martin Urban, Alayne Cuzick, James Seager, Nagashree Nonavinakere, Jahobanta Sahoo, Pallavi Sahu, Vijay Laksmi Iyer, Lokanath Khamari, Manuel Carbajo Martinez, Kim E Hammond-Kosack

**Affiliations:** Protecting Crops and the Environment, Rothamsted Research, Harpenden AL5 2JQ, UK; Protecting Crops and the Environment, Rothamsted Research, Harpenden AL5 2JQ, UK; Protecting Crops and the Environment, Rothamsted Research, Harpenden AL5 2JQ, UK; Molecular Connections, Kandala Mansions, Kariappa Road, Basavanagudi, Bengaluru 560 004, India; Molecular Connections, Kandala Mansions, Kariappa Road, Basavanagudi, Bengaluru 560 004, India; Molecular Connections, Kandala Mansions, Kariappa Road, Basavanagudi, Bengaluru 560 004, India; Molecular Connections, Kandala Mansions, Kariappa Road, Basavanagudi, Bengaluru 560 004, India; Molecular Connections, Kandala Mansions, Kariappa Road, Basavanagudi, Bengaluru 560 004, India; European Molecular Biology Laboratory, European Bioinformatics Institute, Wellcome Genome Campus, Hinxton, Cambridge CB10 1SD, UK; Protecting Crops and the Environment, Rothamsted Research, Harpenden AL5 2JQ, UK

## Abstract

The Pathogen–Host Interactions Database (PHI-base) has, since 2005, provided manually curated genes from fungal, bacterial and protist pathogens that have been experimentally verified to have important pathogenicity, virulence and/or effector functions during different types of interactions involving human, animal, plant, invertebrate and fungal hosts. PHI-base provides phenotypic annotation and genotypic information for both native and model host interactions, including gene alterations that do not alter the phenotype of the interaction. In this article, we describe major updates to PHI-base. The latest version of PHI-base, 4.17, contains a 19% increase in genes and a 23% increase in interactions relative to version 4.12 (released September 2022). We also describe the unification of data in PHI-base 4 with the data curated from a new curation workflow (PHI-Canto), which forms the first complete release of PHI-base version 5.0. Additionally, we describe adding support for the Frictionless Data framework to PHI-base 4 datasets, new ways of sharing interaction data with the Ensembl database, an analysis of the conserved orthologous genes in PHI-base, and the increasing variety of research studies that make use of PHI-base. PHI-base version 4.17 is freely available at www.phi-base.org and PHI-base version 5.0 is freely available at phi5.phi-base.org.

## Graphical Abstract

**Graphical Abstract:**
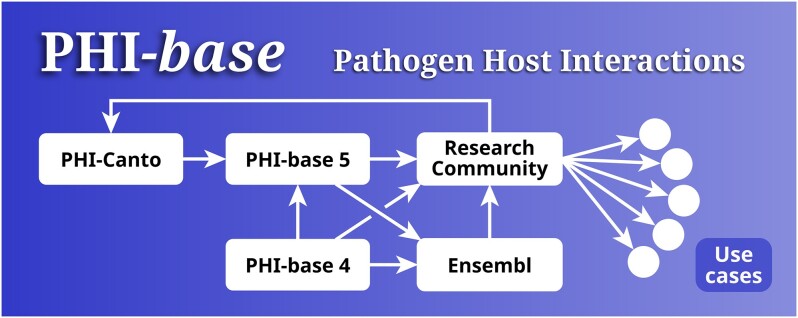

## Introduction

The health of plants, animals, humans and entire ecosystems can be temporarily or persistently compromised by the occurrence of one or more infectious diseases. Globally as well as locally, infectious diseases threaten the economic wealth of continents, countries and regions, human and farmed animal community structures, the availability and expense of foods, feeds and fibres, and the biodiversity of natural and human-modified aquatic and terrestrial ecosystems (1–6). In addition, the incidence, severity and impact of infectious diseases are steadily rising due to increased global travel and the trading of fresh produce. As a result of a changing climate, disease impacts can be further exacerbated through the ever-widening range of detrimental effects, including the co-occurrence of negative biotic and abiotic threats (4,7). In many regions of the world, pathogenic species are migrating poleward (8) or to higher elevations (9). This is primarily caused by the gradual rising in global temperatures and unusual rainfall patterns. As a result, host species are becoming infected with unfamiliar pathogens, and novel disease outbreaks are occurring (10,11). In many agricultural, medical and veterinary settings, the number and type of anti-infective chemicals available commercially that can still be used to control infectious diseases effectively are now highly reduced, either because new legislation has banned or restricted the use of previously registered chemistries or because of the emergence of chemical-resistant species or strains (12,13). Within the ‘One Health’ concept, which evolved through a sequential series of health domain-specific conferences held during the late 2000s (see reviews (14,15)), there is growing recognition of the multiple interconnectedness between crop health, animal health, human health and ecosystem health (14,16–18). In addition, the metagenome of microbial communities associated with plants, animals and humans can be said to function as a ‘second genome’ more commonly referred to as a metagenome that drives the fitness and performance of almost all organisms (11,15). Collectively, as a result of the increased societal, economic and ecological relevance of infectious diseases, there is an ongoing and pressing need to understand the underlying pathogenic processes *per se* and how these processes are evolving in the myriad of disease-causing species, as well as to understand how host species combat very different infection processes and the spectrum of ‘in host’ pathogen lifestyles.

The Pathogen–Host Interactions database (PHI-base), established in 2005, is freely available at www.phi-base.org. PHI-base adheres to the FAIR principles to ensure data is Findable, Accessible, Interoperable and Reusable (19). PHI-base stores expertly curated molecular and biological information on genes proven to affect the phenotypic outcome of pathogen–host interactions (PHIs). Each entry in PHI-base has strongly supported experimental evidence obtained from a peer-reviewed publication. The term ‘interaction’ in each PHI-base entry is used to define the observable function of either one gene or one protein, on one host, and on one tissue type or one *in vitro* situation (20). PHI-base includes entries for bacterial, fungal and protist pathogens which infect plant, human, animal, insect and other hosts, but does not include viral pathogens. The PHI-base entries also cover the direct first-host targets of pathogen effectors (when known) and the targets of commercial anti-infective chemicals. To enhance PHI-base's usage in comparative, pangenome, diagnostic and evolutionary studies, genes experimentally tested but found not to affect the interaction outcome are also curated. In addition, although several features in PHI-base have changed or gradually evolved over the past 20 years, nine high-level phenotypic outcome terms remain in long-term use to permit the comparison of interactions across the entire tree of life (21). These terms are ‘loss of pathogenicity’, ‘reduced virulence’, ‘increased virulence (hypervirulence)’, ‘unaffected pathogenicity’, ‘effector’, ‘lethal’, ‘enhanced antagonism’, ‘resistance to chemical’ and ‘sensitivity to chemical’. In comparative studies, biologists and computational biologists find these high-level terms particularly useful when undertaking multi-discipline analyses or mega-scale data analyses and are often unfamiliar with different pathogen-host systems (pathosystems), but aim to include pathogenic species with different lifestyles, host ranges, environmental requirements and/or niche occupancies in their comparative analyses. Within PHI-base, a BLAST tool (PHIB-BLAST, phi-blast.phi-base.org) is available to permit BLAST queries of modest gene lists (<300 sequences per run) arising from functional genomics, transcriptomics, proteomics and protein–protein interaction experimentation.

The phenotypic data in PHI-base is directly connected to the entries for individual genes within the genomes of plant pathogenic species available within Ensembl Fungi, Ensembl Bacteria and Ensembl Protists (22,23) and displayed within entries for plant-, human- and/or animal-infecting species in FungiDB under the content pane ‘Identify Genes based on Phenotype Evidence’ (24) and within UniProtKB (25) under the content pane ‘Phenotypes & Variants’. PHI-base also reuses resources and ontologies provided by external resources, including PubMed, NCBI Taxonomy (26), UniProtKB (25), Gene Ontology (GO) (27), ChEBI (28) and the Fungicide Resistance Action Committee (FRAC) (www.frac.info). Several other multi-pathogen species databases exist, and these have previously been reviewed and compared in detail (29–31). These include Ensembl Genomes (22), FungiDB (24), PATRIC (32) and SecretEPDB (33), which focus, respectively, on providing genomic data and annotations for microbes, comparative genomics tools and functional information for fungal organisms, information on virulence factors and antimicrobial resistance genes in bacteria and secreted bacterial effectors produced by various animal or plant infecting species. Whereas uniquely, PHI-base consistently describes phenotypes using the same controlled generic vocabulary for a wide spectrum of plant, human, animal and insect PHIs across more than 290 pathogenic species.

In this article, we report on a major increase in PHI-base gene content, and the first full release of PHI-base version 5.0, which combines, under one schema, data from PHI-base 4 with newly curated data supplied by curators and authors using the PHI-Canto community curation tool (available at https://canto.phi-base.org) (34). We also report on deeper integration with the Ensembl project by enabling the importing of interactions, phenotypes and anti-infective resistance annotations from PHI-base 4; improving compliance with the FAIR data principles by using the Frictionless Data framework to add additional metadata to PHI-base 4 datasets and assigning persistent identifiers (a DOI) to each dataset via the Zenodo repository; mapping proteins in PHI-base to orthologous groups across functional categories using the eggNOG database; and an increasing breadth of use cases, including functional gene characterisation, ‘omics’ studies and predictive modelling.

## Results and discussion

### Biological data update

Version 4.17 of PHI-base (released in May 2024 and described in this article) contains 9973 genes, 22 415 PHIs, 295 pathogens, 246 hosts, 548 diseases and 5521 references. The number of genes manually curated for PHIs has increased by 18.6% since version 4.12 (reported in 2022) (35). Bacteria and fungal pathogens provide 96.3% of the PHI phenotype annotations (of which 54% involve bacterial pathogens and 46% involve fungal pathogens), whilst protists, protozoa, nematodes and insects provide 3.4% (Table 1). These species contributions are almost identical to those reported for version 4.12. The ascomycete fungi dominate the fungal pathogen curation with 8699 PHI phenotype annotations and 107 species (88% of all fungal PHI phenotypes), followed by the basidiomycetes with 1119 PHI phenotypes and 12 species (11.4% of all fungal PHI phenotypes). Compared to version 4.12, an additional 4229 PHI phenotype annotations describing experimental data for 1562 genes from 1134 newly manually curated publications are included up to May 2024.

**Table 1.** Summary of pathogen taxonomic groups, interactions and phenotypes within PHI-base version 4.17

Data type	Bacterium	Fungus	Protist	Nematode	Insect	Totals
Number of pathogens	148	120	19	6	2	295
Interactions in total	11755	9823	797	30	10	22415
**PHI phenotypes**						
Loss of pathogenicity	330	861	17	1	0	1209
Reduced virulence	5992	4731	215	17	0	10955
Unaffected pathogenicity	2502	2951	141	0	0	5594
Effector (plant avirulence determinant)	2041	682	373	11	10	3117
Increased virulence (hypervirulence)	859	400	42	1	0	1302
Lethal	18	157	9	0	0	184
Chemical target: resistance to chemical	7	29	0	0	0	36
Chemical target: sensitivity to chemical	6	8	0	0	0	14
Enhanced antagonism	0	4	0	0	0	4

The number of pathogenic species in PHI-base has increased by 17 to a total of 295 pathogens. New species include newly emerging pathogens under intense investigation, such as the bacterial fruit rot pathogen *Paracidovorax citrulli*, and species included in comparative studies (Supplementary Table S1). Within PHI-base, plant pathogens represent ∼53% of the species investigated (Table 2). There are now slightly more curated non-cereal infecting species entries at 6128 compared to cereal infecting species at 5514 and a further 135 entries from species infecting other monocot hosts, including *Allium, Lilium* and *Musa* (banana) species. Tree and woody shrub infecting species provide 1360 plant PHI annotations, involving 51 species (11.6% of the plant PHIs), of which 1135 PHIs are for economically important fruit-bearing species in the genus *Citrus, Malus, Prunus* or *Pyrus*. The three model plant species *Arabidopsis thaliana, Nicotiana benthamiana* and *Nicotiana tabacum* continue to provide ∼5% of the data (1218 PHIs). The number of curated PHI phenotypes for pathogens that infect vertebrates, and their model hosts has increased to 43.4% of the total (9726 PHIs), while 25.6% of annotations (5736 PHIs) come from pathogenic species infecting agricultural, horticultural or viticultural crop species. The emerging pathogen species that have been curated for the first time include *Candida auris, Colletotrichum fructicola, Plasmopara viticola, Vibrio alginolyticus* and *Paracidovorax citrulli* (Supplementary Table S1).

**Table 2.** Summary of the number of host species and interactions within PHI-base version 4.17

Data type	Plant	Vertebrate	Insect	Nematode	Others	Totals
Host species	154	43	30	1	18	246
Interactions in total	11777	8670	1352	487	129	22415
**PHI phenotypes** ^†^						
Loss of pathogenicity	808	354	32	14	1	1209
Reduced virulence	4653	5103	810	301	88	10955
Unaffected pathogenicity	3113	1971	343	137	30	5594
Effector (plant avirulence determinant)	2661	413	33	1	9	3117
Increased virulence (hypervirulence)	396	739	132	34	1	1302
Chemical target: resistance to chemical	30	6	0	0	0	36
Chemical target: sensitivity to chemical	13	1	0	0	0	14
Enhanced antagonism	4	0	0	0	0	4

^†^The ‘Lethal’ high-level phenotype is not included since it is not applicable for host species: this phenotype indicates that a mutation in a pathogen renders the pathogen inviable.

The 30 most annotated pathogen species in PHI-base account for 70.7% of the total PHI data, which is provided by the curation of 7082 genes (Table 3). The species in this top 30 list remain the same as for PHI-base version 4.12 and include 12 plant infecting species, 17 human and/or animal infecting species and one insect infecting species. The filamentous fungal pathogens *Fusarium graminearum* and *Magnaporthe oryzae*, which cause various diseases on staple cereal crops, such as wheat, barley, rice and maize, continue to provide the highest number of PHI entries by both interaction types explored and genes/proteins investigated. Amongst the plant-infecting bacteria, entries for specified and non-specified biotypes within the *Ralstonia solanacearum* species complex, which infect potato and/or a range of other *Solanaceae* species, have increased considerably to 947 interaction entries. For the animal kingdom, the most curated pathogens include the human pathogens *Salmonella enterica, Candida albicans, Escherichia coli, Aspergillus fumigatus* and *Cryptococcus neoformans* (Table 3). Entries for *Toxoplasma gondii*, a protozoan parasite that infects most species of warm-blooded animals, including humans, have increased considerably to 286 interaction entries. Two pathogens that infect both plants and immunocompromised animals are well curated, namely the bacteria *Pseudomonas aeruginosa* (748 PHIs) and the fungus *Fusarium oxysporum* (337 PHIs). The entries for the insect infecting biocontrol species *Beauveria bassiana* have increased considerably to 197 interactions.

**Table 3.** Highly annotated pathogens, interactions and proteins within PHI-base version 4.17

Pathogen	Inter- actions	Proteins*	Loss of pathogenicity	Reduced virulence	Increased virulence	Effector	Unaffected pathogenicity	Lethal	No of host species
*Fusarium graminearum* ^a^ (F)	1851	1340	49	704	14	0	990	94	15
*Magnaporthe oryzae* ^a^ (F)	1656	733	313	750	23	104	465	1	7
*Salmonella enterica* ^b^ (B)	1246	550	20	736	103	145	242	0	15
*Candida albicans* ^d^ (F)	787	390	71	483	62	0	167	4	12
*Escherichia coli* ^b^ (B)	662	304	1	411	48	23	178	1	17
*Pseudomonas aeruginosa* ^c, d^ (B)	748	294	21	417	57	12	241	0	24
*Aspergillus fumigatus* ^b^ (F)	474	275	36	234	29	0	131	42	7
*Cryptococcus neoformans* ^b^ (F)	495	264	62	299	27	0	97	10	9
*Ustilago maydis* ^a^ (F)	452	229	53	233	9	30	127	0	3
*Staphylococcus aureus* ^b^ (B)	687	227	17	425	109	2	133	1	13
*Xanthomonas oryzae* ^a^ (B)	629	227	5	157	29	309	129	0	3
*Pseudomonas syringae* ^a^ (B)	441	185	1	101	9	272	57	1	17
*Fusarium oxysporum* ^c^ (F)	337	163	27	165	18	39	88	0	28
*Botrytis cinerea* ^a^ (F)	571	161	43	315	20	8	183	0	34
*Ralstonia solanacearum* ^a^ (B)	947	153	24	89	4	805	24	1	13
*Xanthomonas campestris* ^a^ (B)	284	144	23	137	13	60	49	2	9
*Beauveria bassian*a^e^ (F)	197	140	5	132	19	2	39	0	14
*Toxoplasma gondii* ^b^ (P)	286	136	9	108	7	38	122	2	5
*Mycobacterium tuberculosis* ^b^ (B)	206	133	3	103	42	3	55	0	6
*Erwinia amylovora* ^a^ (B)	549	125	34	240	55	15	205	0	6
*Streptococcus pneumoniae* ^b^ (B)	206	119	4	137	12	0	47	6	4
*Verticillium dahliae* ^a^ (F)	317	108	15	161	13	32	96	0	17
*Streptococcus suis* ^b^ (B)	254	98	5	197	5	0	43	4	7
*Klebsiella pneumoniae* ^b^ (B)	212	96	5	93	5	0	109	0	5
*Vibrio cholerae* ^b^ (B)	209	93	1	131	16	0	61	0	7
*Listeria monocytogenes* ^b^ (B)	282	88	3	198	22	3	56	0	11
*Acinetobacter baumannii* ^d^ (B)	302	88	0	201	12	1	88	0	6
*Streptococcus pyogenes* ^b^ (B)	219	86	3	138	28	0	48	2	7
*Burkholderia pseudomallei* ^b^ (B	112	67	1	75	3	24	9	0	4
*Nakaseomyces glabratus* ^d^ (F)	231	66	0	128	25	0	77	1	5
TOTALS	15849	7082	854	7698	838	1927	4356	172	330

*Genes were mapped to the latest genome assembly and reference UniProtKB proteome where available.

Symbols indicate: ^a^ plant pathogen, ^b^ animal pathogen, ^c^ pathogen of both plant and animal hosts, ^d^ opportunistic pathogen usually only able to infect immunocompromised humans, ^e^ entomopathogenic fungal species used to control insect pests. Taxon indicated in parenthesis (F) fungus, (B) bacterium and (P) protozoa.

Twelve plant-infecting *Fusarium* species now provide 10.5% of all entries (2348 interactions). Eight of these species infect cereals and contaminate the harvested grains with various mycotoxins that are harmful to humans and animals (36). These mycotoxin-producing species contribute in total 1994 interaction entries and reside within the species complexes *sambucinum* (six species) or *fujikuroi* (three species) (37,38). The other three *Fusarium* species reside within the species complexes *solani* (two species) or *oxysporum* (one species), and cause a range of disease types in various non-cereal monocotyledonous and dicotyledonous horticultural or arable species, and are not known to produce mycotoxins.

There are 22 new host species in PHI-base version 4.17. This includes 16 plant species, 5 fish species and 2 crustacean species as either the natural host(s), or the surrogate model host for testing (Supplementary Table S2). New fish test species include two species each of carp, grouper and Senegalese sole. New plant species include three additional species to test effector functions, as well as two new host–pathogen interactions of emerging concern. New plant species curated for the first time include *Beta vulgaris, Fraxinus excelsior, Panax ginseng, Trifolium pratense* and *Vitis riparia*.

To permit taxonomically wide interspecies comparisons, PHI-base has consistently assigned high-level phenotype annotations to all interaction entries (21). These phenotype annotations are summarised for the pathogen species in Table 1 and for the host species in Table 2. For pathogens, the ‘reduced virulence’ phenotype has the highest number of PHI annotations at 10 955 (48.9%), whereas the ‘loss of pathogenicity’ PHI phenotype has only 1209 entries (5.4%), a split similar to previous releases (35). The ‘loss of pathogenicity’ entries are far more prevalent for the plant pathogens, at 67%. The number of genes with an ‘increased virulence’ PHI phenotype when a pathogen gene is modified or deleted now stands at 1302 entries, a rise of 34% compared to the 2022 figure. More than half of these entries are for bacterial pathogens. For hosts, there has been a considerable rise (34%) in the number of interactions annotated with ‘increased virulence’ for vertebrate infecting pathogens (739 entries). This reflects the continuing efforts from the international research community to identify and inter-compare the negative regulators and feedback control points in different host–pathogen systems.

Since 2016, a major curation effort for PHI-base has been to increase the coverage of pathogen effectors. An effector is an entity derived from a pathogenic or non-pathogenic species, that either activates or suppresses the host's defensive responses or other responses (39–42). The number of curated pathogen effector proteins interacting directly with one or more host species is 830 genes tested in 3117 interactions, an increase since version 4.12 of 26.3% and 18%, respectively. Effectors represent 14% of all interaction entries in PHI-base. Effector entries from 75 plant-infecting pathogenic species provide 86% of the curated interactions, whilst 14% of interaction entries are provided from 33 species infecting human, other vertebrates or invertebrates (Table 4). Most of the plant interactions are contributed by non-cereal infecting bacteria, especially *Ralstonia solanacearum* (805 interactions, 83 genes), various *Xanthomonas* species (469 interactions, 70 genes) and various *Pseudomonas* species (290 interactions, 93 genes). However, the number of fungal species infecting plant species that contribute effector entries has risen considerably from 21 to 37 species, and these interaction entries have risen by 62% to 681. Most fungal effector genes have been explored for the rice blast fungus *Magnaporthe oryzae* (35 genes). Whilst interaction entry numbers are high for the wheat infecting fungus *Pyrenophora tritici-repentis* (138 interactions) and for the fungus *Colletotrichum orbiculare* (65 interactions) which causes disease on melon and cucumber fruit even though only two genes from each species have been investigated. The three model plant species, *Nicotiana benthamiana, Nicotiana tabacum* and *Arabidopsis thaliana* continue to provide a high proportion of the plant effector entries at 660 interaction (25%). Increasingly, in these *in planta* bioassays, research is focused on comparing the induction of non-host responses and the consequences of race-specific interactions between resistance proteins and avirulence proteins and is also reporting on the various subcellular locations targeted by effector proteins, including chloroplasts, nuclei and mitochondria (39,43). Curated effector studies involving human, vertebrate and model invertebrate hosts from bacterial pathogens continue to increase, with total entries now at 379 interactions, 203 genes and 31 species in version 4.17. *Salmonella enterica* still provides by far the highest number of entries (145 interactions, 90 genes), but there are a growing number of entries from *Coxiella burnetii, Legionella pneumophila* and *Acinetobacter nosocomialis*. Also of note for the first time are 13 effector entries from bacterial species from poor-quality water environments that cause diseases in fish species. The bacterial pathogens involved are *Edwardsiella ictaluri* and *Edwardsiella tarda*, which cause either acute septicaemia or chronic encephalitis; *Aeromonas hydrophila*, which enters the blood and causes organ damage by producing a cytotoxic endotoxin; and *Aeromonas salmonicida*, which causes furunculosis, which includes sepsis and haemorrhaging. *Aeromonas hydrophila* is also a well-recognised human pathogen. Thirty-two bacterial effector entries involve five bacterial species of humans and/or vertebrates, which have been tested using the larvae of *Galleria mellonella* (greater wax moth). This curation indicates the further gradual adoption of the 3Rs principles (replacement, reduction and refinement) by the international research community. The new plant host species contributing comparative effector entries are *Actinidia arguta, Beta vulgaris* and *Solanum albicans*.

**Table 4.** Summary of the pathogenic species providing the most information on effectors

PLANT PATHOGENS - 75 species	Interactions - 2698	Proteins - 606
**Bacteria - 22 species**	**1662**	**306**
*Ralstonia solanacearum*	805	83
*Xanthamonas* species	469	70
*Pseudomonas* species	290	93
*Burkholderia pseudomallei*	24	23
*Paracidovorax citrulli*	23	7
*Burkholderia glumae*	15	5
*Erwinia amylovora*	15	10
**Fungus - 37 species**	**681**	**168**
*Pyrenophora tritici-repentis*	138	2
*Magnaporthe oryzae*	104	35
*Colletotrichum orbiculare*	65	2
*Fulvia fulva*	64	9
*Fusarium oxysporum*	39	19
*Verticillium dahliae*	32	9
*Ustilago maydis*	30	16
*Rhynchosporium commune*	21	3
*Zymoseptoria tritici*	17	6
*Ustilaginoidea virens*	15	3
*Leptosphaeria maculans*	15	7
**Obligate biotrophic fungi - 6 species**	**89**	**24**
*Puccinia* species	38	8
*Melampsora* species	36	7
*Blumeria* species	14	8
**Protists - 10 species**	**334**	**124**
*Hyaloperonospora arabidopsidis*	127	45
*Phytophthora sojae*	79	30
*Phytophthora capsici*	57	13
*Phytophthora infestans*	47	24
**Nematode and insects - 6 species**	**21**	**8**
**HUMAN / VERTEBRATE / INVERTEBRATE PATHOGENS - 33 species**	**Interactions - 419**	**Proteins - 224**
**Bacteria - 31 species**	**379**	**203**
*Salmonella enterica*	145	90
*Coxiella burnetii*	46	11
*Legionella pneumophila*	30	15
*Acinetobacter nosocomialis*	30	2
*Escherichia coli*	23	16
*Yersinia pestis*	16	7
*Citrobacter rodentium*	14	7
*Francisella tularensis*	11	7
*Chlamydia trachomatis*	10	4
**Fungi - 1 species** *Beauveria bassiana*	2	2
**Protozoan - 1 species** *Toxoplasma gondii*	38	19

There are growing concerns over the impacts of climate change on agricultural production systems and the transportation of fresh commodities and how these changes will affect global food and feed security. Consequently, researchers across the globe are increasingly focusing on investigating pathogenic interactions involving the current crop plant species, as well as new species anticipated to be grown in new regions, or grown at greater frequencies in the future. The interaction entries in PHI-base involving major food and feed crops continues to rise and includes significant curation for wheat (2106), rice (1815), maize (902), barley (618), tomato (880), potato (175), soybean (207) and *Brassica* species (303). Collectively, the 86 crop plant species provide 10 082 interaction entries, which is 85.6% of the total plant entries and involves 135 pathogenic species. The cereal species provide 5510 interactions and involve 47 pathogenic species, including the fungi *Fusarium graminearum, Magnaporthe oryzae* and *Ustilago maydis* (from the top 30 list) as well as other key global species, including the major wheat pathogens *Puccinia striiformis* and *Zymoseptoria tritici* – with 31 and 100 entries, respectively – and the major rice pathogens *Ustilaginoidea virens* and *Burkholderia glumae*, with 37 and 74 entries, respectively.

### Collaboration with Ensembl Genomes

Since November 2022, we have contributed data on molecular interactions and phenotypes to Ensembl Fungi, Ensembl Bacteria, and Ensembl Protists. More recently, Ensembl developed a new data model (22) to capture interactions between any two entities (proteins, genes, mRNA and small molecules) and built visualisations into their gene pages and REST APIs for querying. In collaboration with the Ensembl team, we developed new tabular formats to distribute datasets on PHIs, gene-for-gene interactions, protein–protein interactions, and interactions between pathogens and antimicrobial chemicals (which describe changes in antimicrobial resistance). While these were primarily aimed at the Ensembl data import pipeline, these are freely available for any other resource to use as input.

As of September 2024, the following subset of PHI-base interactions has been loaded into Ensembl: 5199 interactions from PHI-base 4.17 and 135 interactions from PHI-base 5.0, of which 55 are anti-infective microbial interactions. The Ensembl interactions schema was designed to aggregate relationships involving the same entities (e.g. a protein) in a reference genome into a single record, linked to metadata about the experimental or environmental conditions in which the interaction was observed. Future modifications to disambiguate multiple mutations of the same protein and strain variations as unique records are being discussed, in order to be able to represent the full repertoire of the PHI-base data.

### Conserved orthologous gene analysis of PHI-base

**Figure 1.**
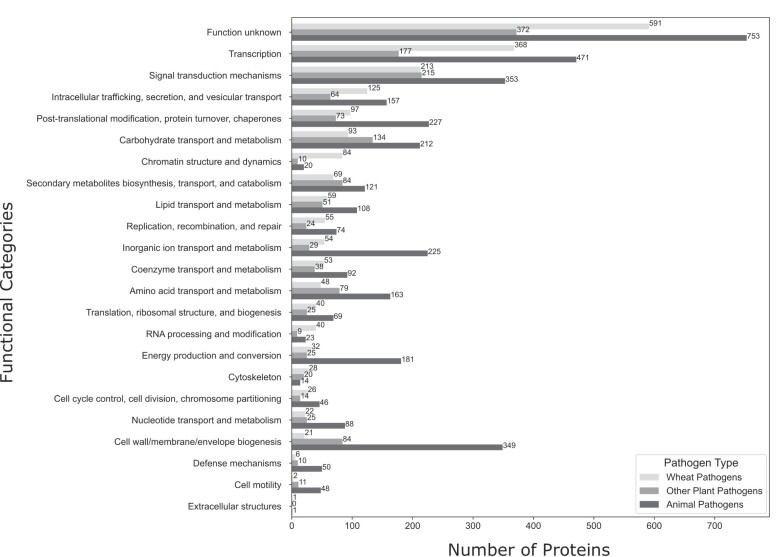
Distribution of proteins across COG categories for wheat, plant (non-wheat) and animal pathogens. The bar plot shows the distribution of mapped PHI proteins (7545 proteins) to EMBL’s EggNOG database (44) for wheat pathogens (2127 proteins), other plant pathogens (1573 proteins) and animal pathogens (3845 proteins) across 23 COG functional categories (*y*-axis). Functional categories are grayscale-coded by pathogen type. The total number of mapped proteins in each functional category is shown on the bars to allow comparison between pathogen groups

To explore the distribution of functional diversity, 7545 out of 9051 PHI proteins were mapped to orthologous groups across different functional categories using the eggNOG database (Release v2.1.9) (44). These proteins were mapped to broad functional categories and grouped by host specificity – ‘animal pathogen’, ‘wheat pathogen’ and ‘other plant pathogen’ – to investigate unique infection mechanisms and where the greatest functional knowledge currently resides. The mapping identifies several trends in functional allocation among the three pathogen groups (Figure 1). Datasets for these three nominated pathogen groups and data transformation scripts are accessible on Zenodo (45). Animal pathogens exhibit a wider diversity of functions, particularly in categories related to cell wall, cell membrane and cell envelope biogenesis, and inorganic ion transport and metabolism, indicating specialised adaptations to their animal hosts. In contrast, wheat pathogens were enriched in proteins associated with chromatin structure and dynamics, suggesting the presence of unique regulatory mechanisms adapted to infect wheat. Other plant pathogens showed diverse functionalities, albeit with fewer specific enrichments compared to the other two categories. A significant portion of the PHI proteins (35%) could not be mapped in the eggNOG database or were categorised as having an unknown function. This observation aligns with a study by Vanni et al. (2022), which reported that about 30% of microbial sequences can not be functionally annotated, reduced from a previously higher reported value of 40–60% of functionally unannotated proteins (46). Additional functional assays and experiments are needed to fill this functional annotation gap.

### Full release of PHI-base 5 and data migration

The first full release of PHI-base 5 (version 5.0) took place in March 2024. This release contains data from 81 publications, curated using PHI-Canto and the pathogen–host interaction phenotype ontology (PHIPO), plus curation from 4686 publications migrated from PHI-base 4 to the new PHI-base 5 schema. The data content is described in Table 5. Of note are the new annotation types, many of which have been used to supply annotations that were previously unrepresented (or underrepresented) in PHI-base 4, such as *Gene-for-Gene phenotype* and *Host phenotype*. The PHI-base 5.0 dataset has been published on Zenodo (47), where it is available both in its source JSON format, and as an Excel spreadsheet for ease of use.

**Table 5.** PHI-base 5.0 data migration statistics

Data type	PHI-Canto	PHI-base 4
Pathogen species	37	275
Host species	23	213
Genes	168	8441
Pathogen genes	118	8417
Host genes	50	0
Interactions (Metagenotypes)	374	27 600
Publications	81	4686
Annotations	1576	38 671
Pathogen–host interaction phenotype	594	15 942
Gene-for-gene phenotype	108	0
Pathogen phenotype	569	7432
Host phenotype	10	0
Disease name	64	14 244
GO biological process	79	1053
GO Cellular component	29	0
GO Molecular function	77	0
Physical interaction	13	0
Post-translational modification	4	0
Wild-type protein expression	2	0
Wild-type RNA expression	27	0

In total, 17 185 records were migrated from PHI-base 4.16, which is approximately 79% of the total number of records. PHI-base 4.17 was not used because data migration preceded its release. Since one record in PHI-base 4 may contain more than one phenotype, each record may generate more than one annotation in PHI-base 5. Consequently, the total number of annotations generated is 38 671, more than double the number of records (Table 5). The remaining 21% of records (4490 records) could not be migrated due to several reasons, the most common being the lack of a valid UniProtKB accession number (2616 records; 12.07% of the total number of records), which is required for compatibility with the schema used for curation in PHI-Canto. The other five reasons, and the number of records excluded because of them, are shown in Supplementary Table S3.

### Updates to the PHI-base 5 website

**Figure 2.**
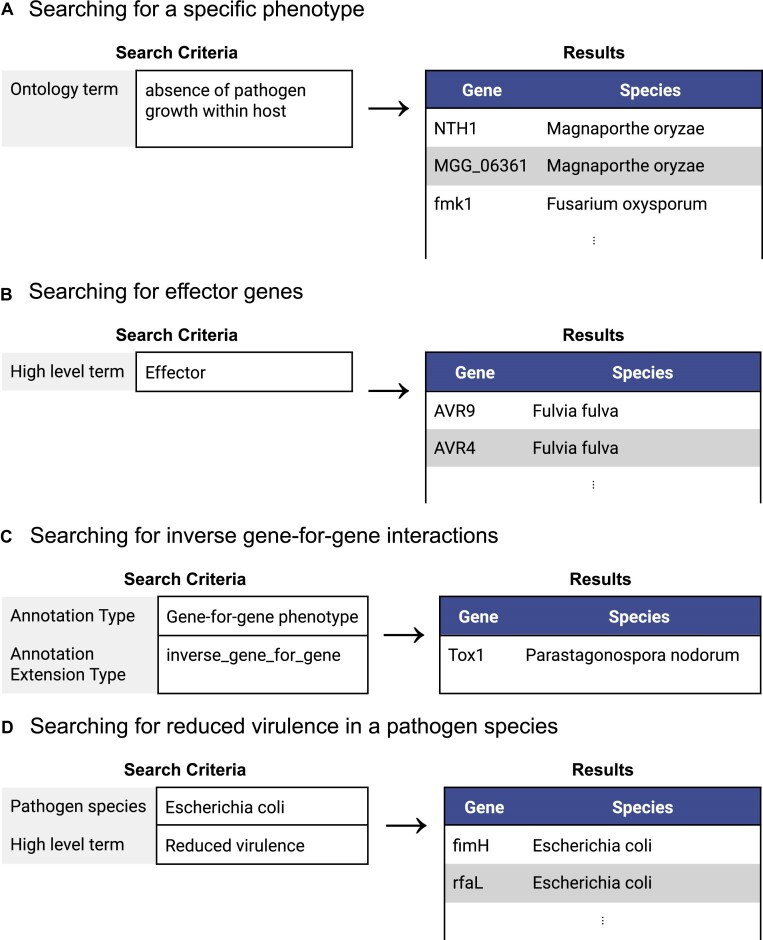
Simplified examples of the Advanced Search feature on the PHI-base 5 website with abbreviated search results. Examples are: (**A**) searching for genes in an interaction that is annotated with a single PHIPO phenotype term; (**B**) searching for genes annotated with the PHI-base 4 high-level term ‘Effector’; (**C**) searching for genes that are part of inverse gene-for-gene interactions; (**D**) searching for *E. coli* genes that are associated with reduced virulence (e.g. when deleted). Vertical dots indicate that search results have been omitted for brevity.

Changes to the PHI-base 5 website since the last NAR article (35) were focused on fixing bugs related to the display and loading of data: over 50 bugs were fixed (48). In particular, multiple issues related to the Advanced Search functionality (https://phi5.phi-base.org/#/search-list-page) were fixed, which now permits searching on a range of data types, including: pathogen or host gene, pathogen or host species, annotation type, the ontology term used for annotation (including disease names), the nine high-level terms used in PHI-base 4, experimental conditions and publication year (for the source publication). Some examples of searches and their results are shown in Figure 2; further examples are shown in Supplementary Figure S1.

The Advanced Search returns a list of genes in PHI-base that match the search criteria. Each gene links to a webpage for the gene that contains all annotations made to the gene or annotations made to interactions involving the gene. Each gene page is divided into sections for each annotation type, with annotations grouped by their ontology term, then by the biological feature that was annotated with the term. Biological features include genes, genotypes and *metagenotypes* (a combined pathogen and host genotype that represents the interaction). Expanding an annotation shows additional information, including experimental evidence, experimental conditions and various secondary annotations, such as the host tissue infected and the change in infective ability of the pathogen (for interactions), or the severity of the phenotype (for phenotype annotations on individual organisms).

### New FAIR release formats for PHI-base 4

Starting in September 2021, we have uploaded datasets from PHI-base 4 to the Zenodo data repository (https://zenodo.org/) with the aim to increase compliance with the FAIR principles (19,49). Specifically, *Findability* is increased because Zenodo assigns a persistent DOI to each dataset and indexes the dataset to enable ease of discovery. We chose not to upload versions prior to 4.12, because later releases include all data from earlier versions, and because data errors had been fixed in later versions.

As of August 2024, datasets from PHI-base 4.12 to PHI-base 4.17 have been uploaded to Zenodo. Datasets from version 4.13 and later make use of the Data Package schema, which is part of the Frictionless Data framework (https://frictionlessdata.io/) (50). This was done to better satisfy the FAIR principles of *Reusability* and *Interoperability*. Specifically, *Reusability* is increased due to the Data Package schema being a domain-relevant standard that provides rich metadata, including data usage licences. *Interoperability* is increased by the Frictionless Framework providing a Python library (frictionless-py) that provides an easy way for data consumers to extract and analyse the data for their own purposes.

The publishing of these datasets on Zenodo was assisted by the development of a new data pipeline written in Python, called *phi4pipeline* (51). This pipeline handles the generation of metadata and readme files – both for Zenodo and the Frictionless Data Package – and applies cleaning and validation to the dataset before upload.

### An increasing breadth of use case studies

PHI-base's curated PHI data are used in a range of research studies. Since going online in 2005, PHI-base has been cited 943 times in the scientific literature. A complete list of these references can be found in the ‘About Us’ section for the PHI-base website.

To evaluate the impact and application of PHI-base, we reviewed studies that cited database use between 2020 and 2024. This review highlights the diverse range of PHI-base use cases and can guide future researchers in how to integrate PHI-base data into their studies. Our analysis covered 493 research papers that cited usage of PHI-base version 4, representing various PHIs and environmental niches. PHI-base data were applied in tool development, bioinformatics research and many other fields. The studies are distributed across different environments: crops (44%), human pathogens (16%), veterinary pathogens (6%), water (1%) and other natural and human modified niches (31%).

From these articles, seven major use case categories were identified. These categories are:

- *Reviews and Resource Comparisons* – Several studies involve comparisons of microbial databases and/or descriptions of genomic resources for pathogenic and non-pathogenic species. For instance, Li et al. (52) reviewed and compared the contents, construction methods, and functionalities of nine bacterial databases to guide researchers in selecting appropriate resources for their studies (52). A comprehensive review on advances in genomics, phylogenomics and proteomics resources and methods for *Ascomycota* fungi is provided by Muggia et al. (2020) (53).
- *Pathogen Functional Gene Characterisation* – Many studies have focused on pathogen gene characterisation through gene deletion and modification, particularly in genera and species, such as *Fusarium, Magnaporthe oryzae* and *Botrytis cinerea*. For instance, Du et al. (54) compared *Metarhizium anisopliae* and *Metarhizium acridum*, revealing that *M. anisopliae* has 98 more virulence-related proteins, aiding host penetration and adaptation, along with differences in secondary metabolite gene clusters that affect pathogenicity (54). Wernet et al. (55) identified the small-secreted protein CyrA in the nematode-trapping fungus *Duddingtonia flagrans* as being crucial for nematode capture, with deletion strains showing reduced virulence (55).
- *Omics Studies* – PHI-base data play a crucial role in ‘omics’ research, including whole genome analysis, phenotypic annotation, functional annotation of transcriptomics, proteomics, and/or metagenomics datasets. These studies also include a number of research articles on secondary metabolite clusters. Genome sequencing centres use PHI-base FASTA data to include phenotype annotations in their annotation pipelines. Researchers apply PHI-base to large-scale comparative genomics and pangenome analyses. For example, Amezrou et al. (2024) identified 19 candidate genes linked to quantitative pathogenicity and host adaptation in *Zymoseptoria tritici* using a multi-host genome-wide association study (56). Alouane and colleagues (2021) sequenced eight *Fusarium graminearum* strains with diverse virulence phenotypes, and identified over 900 secreted protein clusters involved in host adaptation and aggressiveness (57).
- *Microbial Discovery and Characterisation* – PHI-base supports the annotation of novel genomes and the assessment of their pathogenic potential. For example, Liu (58) used PHI-base to characterise the halophilic Archaea *Haloterrigena* obtained from salt lake environments (58). Vannier et al. (59) used metatranscriptomics to identify over 3000 microbial genes involved in microbiota colonisation of plant roots within synthetic microbiota (59). Here, the PHI-base PHIB-BLAST tool was used to identify homologues of differentially expressed fungal genes that had been previously functionally characterised.
- *Environmental and Niche Studies* – PHI-base data are utilised in research focused on pathogens residing within various environmental niches, including natural aquatic systems, crop ecosystems and human pathogens in clinical settings. For instance, in freshwater systems, the blue-green alga *Microcystis* produces microcystin toxin, which is speculated to contribute to the spread of antibiotic microbial resistance genes by inducing stress responses and mutations in *Pseudomonas, Proteobacteria*, and *Bacteroidetes* (60). Airborne pathogen detection is another emerging field in which researchers sequence the DNA (Air-seq) and/or RNA content of air samples for species-level identification. Giolai et al. (61) demonstrated Air-seq's ability to track agriculturally significant pathogens in crop fields and correlated their abundance with environmental factors such as weather (61). PHI-base data were used in this study to confirm the presence of a wheat pathogen. Similarly, Nunez et al. (62) examined bioaerosols in hospitals and found pathogenic bacteria and fungi in both indoor and outdoor samples (62).
- *Host-Pathogen Interaction and Predictive Models* – PHI-base data are often used to predict host–pathogen interactions and develop computational models to study these relationships. Liu et al. (63) employed a weighted gene co-expression network analysis (WGCNA) to identify gene modules and hub genes involved in *Sporisorium scitamineum* infection of sugarcane (63). Of the 83 identified PHI-base homologues, 62 were linked to pathogen virulence, highlighting the use of PHI-base in understanding early infection mechanisms. Guerrero-Egido et al. (64) developed bacLIFE, a computational workflow for predicting lifestyle-associated genes (LAGs) in bacteria. Their analysis of 16846 bacterial genomes identified key LAGs involved in plant-pathogenic lifestyles which were confirmed by plant bioassays and PHI-base data (64).
- *Bioinformatics Tools and Computational Platforms* – Several articles describe and discuss the development of novel computational tools and platforms that use PHI-base to provide PHI data. Wang et al. (65) developed BastionHub, a universal platform integrating and analysing substrates secreted by Gram-negative bacteria. BastionHub incorporates multiple secretion systems and provides tools for substrate prediction and analysis (65). Rozano et al. (66) used the effector proteins stored in PHI-base to assess template-based modelling for its ability to model the structure of fungal candidate effector proteins obtained from bioinformatics analysis (66).

In summary, PHI-base's curated PHI data are used in an increasing variety of research studies from gene characterisation, ‘omics’ studies, new microbial species annotation, environmental studies, to bioinformatics tool development.

## Future plans

Within a year from the date of this publication, we aim to migrate as much as possible of the remaining 22% of records not yet migrated from PHI-base 4 into PHI-base 5. Any records that cannot be migrated will be kept in the PHI-base 4 releases on Zenodo, and a list of any unmigrated records will be included in future PHI-base 5 dataset releases.

Following the release of PHI-base version 4.19, scheduled for May 2025, and after completing the data migration process, all future data releases will adopt the PHI-base 5 schema, and the PHI-base 5 website will become the main website for accessing PHI-base. This means that www.phi-base.org will direct users to the PHI-base 5 website, while the PHI-base 4 website will be archived on a subdomain (provisionally named phi4.phi-base.org). The current subdomain for PHI-base 5 (phi5.phi-base.org) will redirect users to the top-level domain.

We aim to complete the development of, and publish, an automated data pipeline for PHI-base 5, which will handle the release and validation of future datasets. We also aim to add support for the Frictionless Data Package Schema to the PHI-base 5 dataset. Furthermore, we plan to implement cross-linking between the NCBI database and PHI-base in 2025, allowing users to access PHI phenotype data directly from relevant NCBI gene records. This link-out feature will enhance data connectivity, potentially reaching new user groups who may not be familiar with PHI-base. In addition, we intend to explore any possibility to increase the amount of PHI-base annotation that can be displayed in the Ensembl databases, including Ensembl Plants and Ensembl Vertebrates, such as adding support for the display of multiple mutations and variation in the pathogen or host strain. This is particularly relevant to annotation entries for the first direct host targets of pathogen effectors.

The curation of anti-infective chemistry is predicted to rise considerably in the near future, driven by the community's use of the new curation workflow in PHI-Canto (34) and through outreach to specific journal editors to encourage inclusion of data deposition and annotation by authors during manuscript submission. To ensure peer-reviewed articles are fully in-scope for curation, authors will need to deposit the promoter and gene sequences for the known chemistry target site for each strain investigated and phenotyped into a public repository. This additional information will make strain-specific differences in the interaction outcome directly evident through the gene-centric display in PHI-base 5. It will also support growing national and international efforts to identify the cohort of strains suspected of harbouring second-site mutations that contribute to increased resistance to multiple antimicrobial chemistries.

In the long term, we aim to investigate, with our collaborators and other researchers, ways to enhance PHI-base with recommender systems, machine learning and artificial intelligence methods (67). These methods will be used to assist our manual curation process in various ways, such as automating literature triage, extraction of gene names, ontology terms, and other entities of interest from publications, and derivation of annotations from figures (images) and tables. To assist PHI-base users, we are exploring the addition of conversational search to a future PHI-base version, and a personalisation system where users can be notified of any newly curated information based on their research interests.

We expect that during 2025 – the 20th anniversary year of PHI-base – we will pass the milestone of 10 000 genes curated. We predict that this continually increasing wealth of new knowledge, arising from manually curating the peer-reviewed host–pathogen interactions literature, for such a diverse repertoire of plant, animal, human and invertebrate infecting species, will prove invaluable for multiple new types of investigations. For example: with regard to One Health when investigating inter-kingdom dependencies; with regard to climate change when exploring short and longer-term impacts and co-dependencies on pathogen genome evolution; and for new types of network analyses driven by machine learning, to delve deeper into the mechanisms that control pathogenicity, disease formation, host resistance or antimicrobial resistance.

## Supplementary Material

gkae1084_Supplemental_Files

## Data Availability

PHI-base data are freely accessible through multiple platforms. PHI-base 4 datasets can be accessed via the PHI-base 4 website at http://www.phi-base.org and archived on Zenodo at DOI 10.5281/zenodo.5356870. PHI-base 5, the most recent version with improved functionality, is available at https://phi5.phi-base.org and its corresponding datasets are archived on Zenodo at DOI 10.5281/zenodo.10722192. Researchers can query PHI data through PHIB-BLAST at http://phi-blast.phi-base.org and curate data using the PHI-Canto interface at https://canto.phi-base.org. Phenotypic data is mapped to the PHIPO ontology, available at https://obofoundry.org/ontology/phipo.html. Additional PHI-base data is integrated with external platforms such as Ensembl Genomes, available at https://ensemblgenomes.org. KnetMiner is accessible at https://knetminer.com, FungiDB at https://fungidb.org/fungidb/app, and UniProtKB at https://www.uniprot.org. PHI-base code and resources can be accessed at https://github.com/PHI-base. The functional annotation scripts using eggNOG-mapper are available on GitHub at https://github.com/PHI-base/FunCat and archived on Zenodo with DOI 10.5281/zenodo.13730172. The phi4pipeline is available on GitHub at https://github.com/PHI-base/phi4pipeline and Zenodo with DOI 10.5281/zenodo.13773740.
