# Supplementary material for: PHI-base – the multi-species pathogen–host interaction database in 2025
Source: Nucleic Acids Res. 2024 Nov 26;53(D1):D826–38. doi: 10.1093/nar/gkae1084 (PMC11701570; doi:10.1093/nar/gkae1084)
Supplement: gkae1084_Supplemental_Files [file gkae1084_supplemental_files.zip › Supp Fig 1 Advanced Search results.pdf]

Supplementary Figure 1: PHI-base 5 advanced search feature, Examples E to G.

Examples are: (**E**) searching for genes in an interaction annotated with a specific PHIPO phenotype, with a specified experimental variable (high temperature); (**F**) searching for genes that are associated with less virulent forms of pneumonia (where the gene is either wild-type or mutated); (**G**) searching for genes in wild-type interaction phenotypes that are used as control cases for mutant interaction phenotypes; (**H**) searching for genes in all publications published in a single year. Vertical dots indicate that search results have been omitted for brevity.

E. Searching by experimental condition

Search Criteria

Ontology term	decreased rate of hyphae formation
Experimental condition	high temperature

→

Results

Gene	Species
sdhB	Corynespora cassiicola
SdhD	Corynespora cassiicola
⋮	

F. Searching by disease

Search Criteria

Disease	pneumonia
High level term	Reduced virulence

→

Results

Gene	Species
omp38	Acinetobacter baumannii
rcsB	Klebsiella pneumoniae
⋮	

G. Searching by phenotype with wild-type controls

Search Criteria

Annotation type	Pathogen-host interaction phenotype
Annotation Extension type	compared_to_control

→

Results

Gene	Species
See1	Botrytis cinerea
Erg27	Botrytis cinerea
⋮	

H. Searching by publication date

Publication year	Single	2010

→

Gene	Species
AVR-Pita1	Magnaporthe oryzae
popW	Ralstonia solanacearum
⋮	
